# Octahedral tilting in halide double perovskites: disentangling lone-pair chemistry and geometric effects

**DOI:** 10.1039/d6fd00014b

**Published:** 2026-07-23

**Authors:** Mehmet Baskurt, Erik Fransson, Madeleine Lindvik, Paul Erhart, Julia Wiktor

**Affiliations:** a Department of Physics, Chalmers University of Technology SE-412 96 Gothenburg Sweden julia.wiktor@chalmers.se

## Abstract

Halide double perovskites (HDPs) have emerged as promising alternatives to their lead-based counterparts. However, their structural dynamics is less explored than that of conventional halide perovskites. In this work, we investigate octahedral tilting at 0 K and the relative stability of tetragonal and cubic phases of a set of 57 HDPs. By combining structural and energetic descriptors with simple geometric metrics, we identify the main trends controlling the stabilization of one-tilt tetragonal phases across this family. We find that both the magnitude of the tilt angles and the energetic preference for tilted phases correlate primarily with the Goldschmidt tolerance factor *t*. The presence of ns^2^ lone-pair cations also correlates with enhanced tilting; however, this trend largely reflects that lone-pair chemistries in HDPs occur together with ionic sizes that shift *t* away from unity. Consistent with this picture, we observe several compounds without lone pairs that nonetheless exhibit strong octahedral tilting. Finally, using machine-learned interatomic potentials, we connect the 0 K tilting energetics to finite-temperature behavior: compounds with more strongly stabilized tilt phases exhibit higher transition temperatures, and phonon spectra at 350 K reveal soft and broad modes that are consistent with the trends in tolerance factors, tilt angles, and tilt energies at 0 K. Our results provide a systematic reference for structure–stability relationships in HDPs and clarify that lone-pair chemistry is correlated with, rather than the primary cause of, octahedral tilting.

## Abstract 2

We investigate octahedral tilting across 57 halide double perovskites and show that the stability of tetragonal phases is controlled primarily by geometric mismatch, while lone-pair chemistry plays a largely correlated rather than independent role.

## Graphical abstract

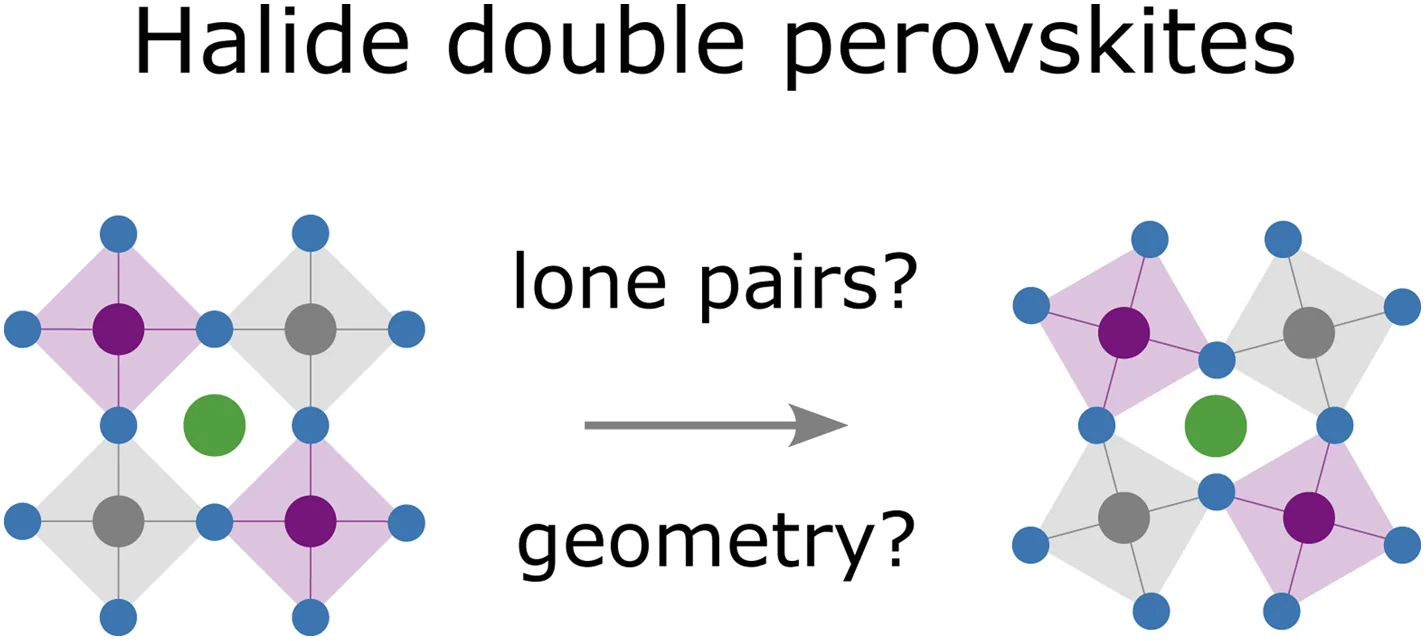

## Introduction

Halide perovskites, known for their exceptional optoelectronic properties, have attracted significant interest, especially for photovoltaic applications.^1–6^ However, concerns surrounding the toxicity of lead-based halide perovskites, as well as stability issues, have spurred an intense search for alternative compositions.^7–10^ Halide double perovskites (HDPs) have emerged as promising lead-free alternatives.^11–14^ Compared to lead-based single perovskites, HDPs offer several advantages, including reduced toxicity and, in many cases, enhanced structural and environmental stability.^15,16^ In addition, the chemical flexibility of HDPs enables access to a wide range of compositions with tunable electronic structures.^17–19^

Beyond composition, lattice dynamics play a crucial role in determining the optoelectronic performance of halide perovskites. Different HDPs can exhibit markedly different degrees of dynamical disorder.^20,21^ While some HDPs have been reported to display reduced anharmonicity relative to lead-based halide perovskites,^22^ other compounds show pronounced anharmonic behavior.^23^ Notably, even closely related halide perovskites can exhibit qualitatively different expressions of anharmonicity.^22^ This motivates a systematic study across the HDP chemical space to identify the factors governing lattice softness and structural dynamics.

Octahedral tilting instabilities play an important role in the structural and electronic properties of HDPs.^24^ Although many halide perovskites adopt a cubic phase at high temperatures, the transition from cubic to lower-symmetry phases, often tetragonal, can occur upon cooling. Such transitions can influence the bandgap, electronic density of states, and carrier mobility, thereby complicating materials design.^24,25^ Therefore, it is crucial to better understand the structural and dynamical properties of HDPs.

Significant efforts have been dedicated to investigating the factors that contribute to the remarkable performance and structural dynamics of halide perovskites. One factor that has been proposed to contribute to structural softness and instabilities is the electron configuration of the octahedral cations. In particular, cations with an ns^2^ electron configuration possess lone-pair electrons that can be stereochemically expressed, and such expression has been discussed as a possible contributor to structural distortions and anharmonicity.^26,27^ At the same time, octahedral tilting is also strongly affected by geometric factors such as ionic size and tolerance-factor arguments,^28^ and tilting distortions can occur even in compositions without lone-pair cations.^21,29^ Disentangling correlation from causation is therefore important when assessing the role of lone-pair chemistry across different materials families.

**Fig. 1:**
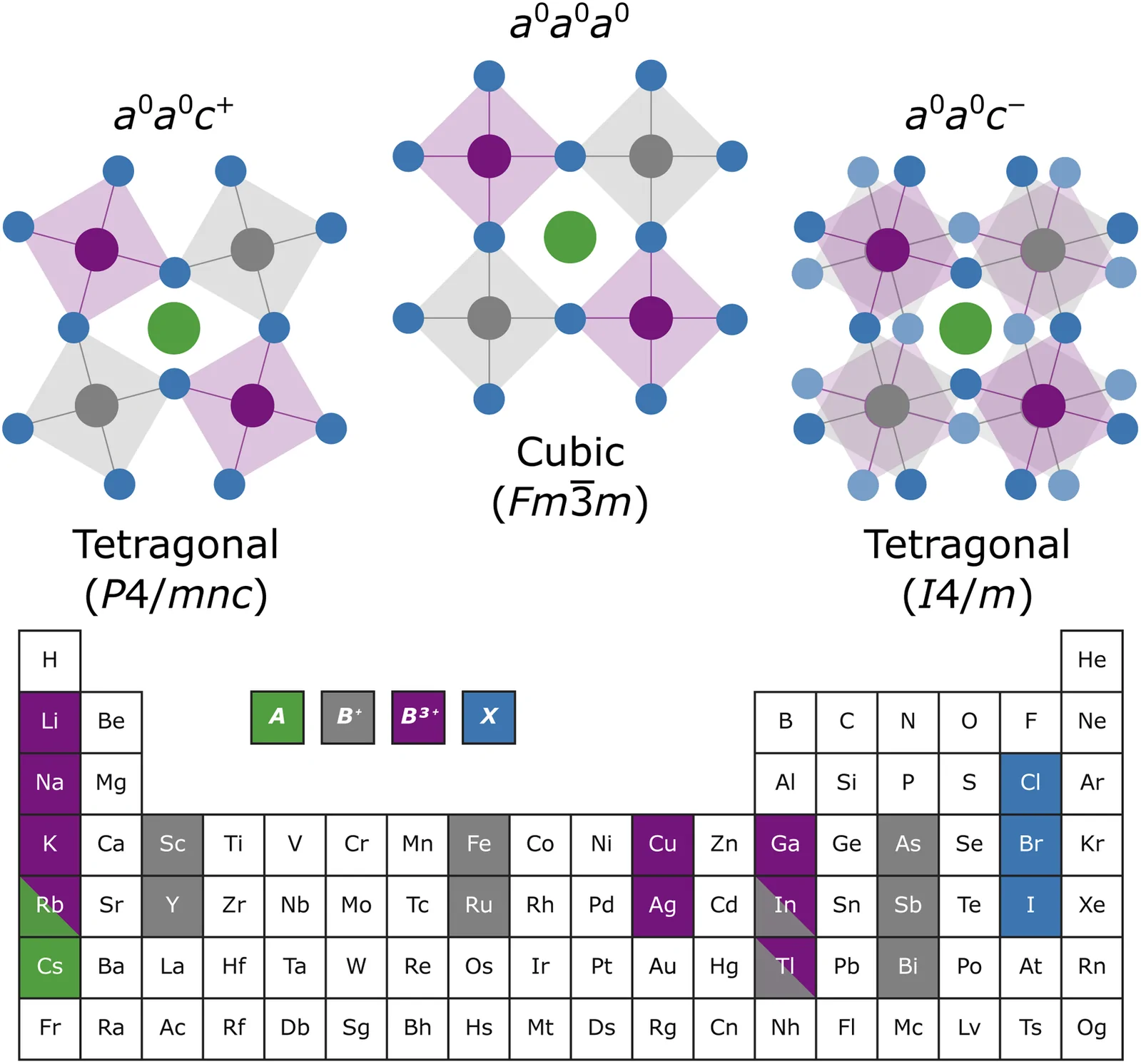
Overview of the double-halide perovskite structures considered in this work. Schematic crystal structures are shown for the tetragonal *P*4/*mnc* (a^0^a^0^c^+^), cubic *Fm*3̄*m* (a^0^a^0^a^0^), and tetragonal *I*4/*m* (a^0^a^0^c^−^) phases. The lower panel maps the chemical space explored, highlighting the selected A, B′/B″, and halide (X) components.

Here, we employ first-principles density functional theory (DFT) to systematically investigate octahedral tilting and phase stability in a set of 57 HDPs spanning a wide range of chemistries and tolerance factors (Fig. 1). By optimizing cubic and symmetry-distinct tetragonal cells, we evaluate octahedral tilt angles and the relative stability of the relevant phases. We then relate these quantities to both the Goldschmidt tolerance factor *t* and the presence of lone-pair cations. Furthermore, using machine-learned interatomic potentials (MLIPs) based on the neuroevolution potential (NEP) framework, we perform molecular dynamics (MD) simulations to study phase transitions in representative materials and connect the 0 K tilting energetics to finite-temperature behavior, including phonon spectral energy density (SED) analysis at 350 K. By using HDPs as a chemically diverse yet structurally consistent platform, our work provides a systematic reference for structure–stability relationships and clarifies that lone-pair chemistry is correlated with, rather than required for, octahedral tilting in halide perovskites.

## Methods

### DFT calculations

We performed first-principles calculations within DFT to investigate the structural properties of HDPs using the Vienna *ab initio* Simulation Package.^30,31^ We employed the SCAN meta-GGA functional^32^ together with the revised rVV10 nonlocal van der Waals correction (SCAN + rVV10) using the parameterization of Peng *et al.*^33^ We note that the choice of exchange–correlation functional can influence predicted lattice parameters, tilt magnitudes, and relative phase energetics in halide perovskites.^34–36^ For halide single perovskites, SCAN + rVV10 has been shown to yield phase transition temperatures in good agreement with experiment.^34^ Calculations were carried out for the cubic (*Fm*3̄*m*) and tetragonal (*I*4/*m* and *P*4/*mnc*) phases of HDPs. For the 0 K structural optimizations and energy differences reported in this work, we used a plane-wave energy cutoff of 720 eV and a Γ-centered *k*-point mesh generated using a reciprocal-space spacing of 0.15 Å^−1^. Structural optimizations were performed allowing full relaxation of atomic positions and lattice parameters. Additionally, DFT calculations were performed to generate the input and training dataset for construction of MLIPs. For this purpose, we employed a reduced cutoff of 520 eV and a *k*-point spacing of 0.25 Å^−1^ to enable efficient sampling of large configuration sets.

In the following, octahedral tilting patterns are described using Glazer's notation.^37,38^ The symbol (a^0^a^0^a^0^) represents the cubic structure, characterized by the absence of octahedral rotations. A pattern of the form (a^0^a^0^c^−^) indicates rotations of the octahedra about the *c* axis with alternating phase in successive layers, while (a^0^a^0^c^+^) corresponds to rotations with the same phase in successive layers. Although two-tilt phases such as orthorhombic or monoclinic exist in various halide perovskites, we limit our study to zero- and one-tilt rotation phases to simplify the comparison between different materials.

The tilt energies were calculated from the energy difference between the cubic (*Fm*3̄*m*) and the tetragonal (*I*4/*m* and *P*4/*mnc*) structures as

1 Δ*E*^tilt^_tetragonal_ = *E*_tetragonal_ − *E*_cubic_.

Here, *E*_cubic_ and *E*_tetragonal_ are the total energies of the optimized cubic (*Fm*3̄*m*) and tetragonal (*I*4/*m* or *P*4/*mnc*) cells, respectively.

For all considered HDPs, we calculated the Goldschmidt tolerance factor:^39^2
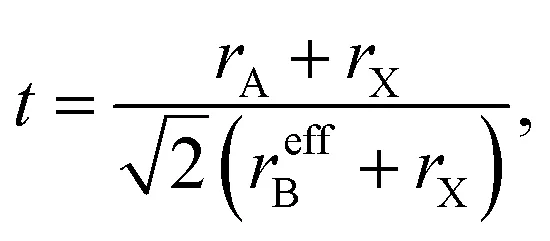
where *r*_A_ and *r*_X_ are the ionic radii of the A-site cation and halide anion, respectively. For double perovskites A_2_B′B″X_6_, the effective B-site radius was taken as the arithmetic average:3
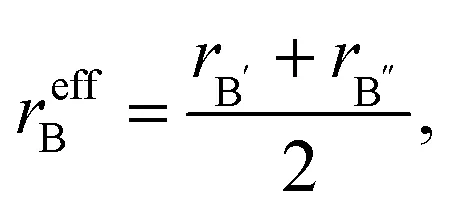
with *r*_B′_ and *r*_B″_ being the ionic radii of the two octahedrally coordinated B-site cations. Ionic radii were mostly taken from Shannon's revised effective ionic radii,^40^ using coordination numbers corresponding to the perovskite environment (12-fold for A, 6-fold for B′/B″, and 6-fold for X). For In^+^, we estimated the radius to be 1.27 Å for CN = 6 by extrapolating the values reported for CN = 9 and 10 by Baloch *et al.*^41^

### Tilt angle analysis

To analyze the octahedral tilting, we extracted tilt angles of B′X_6_ and B″X_6_ octahedra using the implemented functions of the Ovito package.^42^ First, all six B′–X and B″–X bonds were identified in each octahedron. Next, using the algorithm described by Larsen *et al.*,^43^ B′X_6_ and B″X_6_ octahedra were matched to a simple cubic environment, which would occur in the ideal cubic phase of a perovskite crystal, yielding the scaling and rotation necessary for optimal mapping. The quaternion form of the rotation was translated into Euler angles using the SciPy package.^35,44^ In the following, we report, for each structure, the larger of the two tilt angles obtained for the B′X_6_ and B″X_6_ octahedra.

### MLIP construction

We constructed MLIPs using the fourth-generation NEP (NEP4) scheme^45,46^ as implemented in the GPUMD package.^47,48^ NEP models were trained for three representative HDPs, Cs_2_AgAlBr_6_, Cs_2_AgBiBr_6_, and Cs_2_InBiBr_6_. Training datasets were generated using an iterative active-learning workflow.^34^ Candidate configurations were sampled from finite-temperature MD in relevant phases, including cubic and one-tilt tetragonal variants, and selected structures were labeled using DFT. Model uncertainty was estimated using a committee model consisting of one NEP trained on the full dataset and five additional models trained on independent 80/20 train/validation splits; the spread in predicted energies and forces was used to select new configurations. For Cs_2_InBiBr_6_, configurations from the identified monoclinic *P*2_1_/*c* ground-state phase were also included. Training was performed with a radial cutoff of 8 Å and an angular cutoff of 4 Å, for 300 000 generations.

### MD simulations

We performed MD simulations using the GPUMD package.^47,48^ Simulations were carried out in the NPT ensemble using the stochastic cell rescaling barostat.^49^ Starting from the cubic structure, the temperature was decreased from 500 K to 10 K using a time step of 5 fs. After an initial equilibration of 2.5 ns at 500 K, the system was continuously cooled over 120 ns. Simulation cells consisted of 40 000 atoms, corresponding to a 10 × 10 × 10 supercell of the conventional cubic unit cell (40 atoms).

### Spectral energy density

To assess the phonon dispersion at finite temperature, we evaluated the SED from MD simulations. We used supercells consisting of 24 × 24 × 24 repetitions of the primitive cubic unit cell (10 atoms). The simulations were first equilibrated for 100 ps in the NVT ensemble, using the corresponding lattice parameter obtained from NPT simulations, after which the trajectory was sampled every 25 fs in the NVE ensemble. The SED was then computed from the resulting trajectory using Dynasor.^50,51^

## Results and discussion

To establish structure–stability relationships across the HDP family, we investigate the tilt energies, Δ*E*^tilt^_phase_, and tilt angles, *θ*, of symmetry-distinct one-tilt tetragonal variants (*I*4/*m* and *P*4/*mnc*) relative to the cubic phase. Restricting the analysis to zero- and one-tilt phases enables a consistent comparison across compositions and provides a uniform reference for assessing trends in tilting energetics and geometry. The tilt energies are calculated with respect to the cubic phase.

First, we generate primitive cells for the cubic (*Fm*3̄*m*) and two symmetry-distinct tetragonal tilt variants (*I*4/*m* and *P*4/*mnc*) of HDPs A_2_B′B″X_6_. We considered A = Cs and Rb and X = Cl, Br and I. The B′ site is restricted to chemically plausible monovalent cations (Li, Na, K, Rb, Cu, Ag, In^+^ and Tl^+^), while B″ includes representative trivalent cations spanning d^0^/d^10^ and ns^2^ chemistries (Sc, Y, Fe, Ga, In^3+^, Tl^3+^, As, Sb and Bi). We select compositions that systematically span a wide range of ionic size mismatch, tolerance factors, and lone-pair chemistry in order to assess trends in octahedral tilting energetics and angles.

Next, we carry out DFT calculations to optimize the structures of HDPs with initial tilts (a^0^a^0^a^0^), (a^0^a^0^c^−^), and (a^0^a^0^c^+^) by relaxing both cell parameters and atomic positions. We determine the tilt energies and tilt angles as described above. HDPs with corresponding tilt energies of the tetragonal *I*4/*m* and *P*4/*mnc* phases, Δ*E*^tilt^_*I*4/*m*_ and Δ*E*^tilt^_*P*4/*mnc*_, respectively, are given in Table 1. To aid interpretation, the number of ns^2^ lone pairs (LP) per formula unit is also included.

**Table 1:** Number of ns^2^ lone pairs (LP), relative energies of the tetragonal phases (per formula unit), octahedral tilt angles, and Goldschmidt tolerance factor *t* for halide double perovskites. Entries are grouped by LP and sorted by decreasing *t* within each group. For structures close to the cubic limit, the energy landscape is shallow and small tilt angles and energies are sensitive to numerical settings. These values should therefore be interpreted qualitatively

Material	LP	Δ*E*_*I*4/*m*_ (meV f.u.^−1^)	Δ*E*_*P*4/*mnc*_ (meV f.u.^−1^)	*θ*_*I*4/*m*_ (°)	*θ*_*P*4/*mnc*_ (°)	*t*
Cs_2_LiInCl_6_	0	0.08	5.19	0.20	0.00	1.01
Cs_2_NaFeCl_6_	0	0.23	−7.37	0.07	0.03	0.99
Rb_2_LiScCl_6_	0	0.30	5.58	2.67	1.62	0.97
Cs_2_AgAlBr_6_	0	−0.07	−3.15	0.27	0.20	0.97
Rb_2_LiInCl_6_	0	−6.85	−4.87	7.37	5.37	0.96
Cs_2_AgFeCl_6_	0	−0.93	−6.46	0.02	0.27	0.96
Cs_2_NaInCl_6_	0	0.06	−3.19	0.03	0.07	0.96
Cs_2_AgGaBr_6_	0	−0.09	1.69	0.08	0.08	0.95
Cs_2_AgScCl_6_	0	0.17	2.89	0.14	0.01	0.95
Cs_2_NaInBr_6_	0	−0.01	−1.95	1.37	1.36	0.95
Cs_2_NaTlCl_6_	0	0.57	3.71	1.60	0.80	0.94
Cs_2_AgInCl_6_	0	−0.05	0.65	0.01	0.00	0.94
Cs_2_AgScBr_6_	0	−0.15	1.54	0.40	0.32	0.93
Cs_2_AgInBr_6_	0	−1.69	1.70	1.55	1.39	0.93
Cs_2_AgYCl_6_	0	2.92	2.41	3.49	2.31	0.92
Rb_2_NaInCl_6_	0	−42.76	−41.72	11.43	10.94	0.92
Cs_2_AgTlBr_6_	0	1.05	2.91	1.15	0.20	0.91
Rb_2_NaInBr_6_	0	−129.70	−121.21	13.45	13.45	0.91
Cs_2_KInCl_6_	0	−20.58	−12.84	9.93	8.91	0.90
Cs_2_AgYI_6_	0	2.63	2.44	3.37	2.30	0.89
Cs_2_NaSbCl_6_	1	−1.29	1.45	1.34	1.56	0.97
Cs_2_LiBiCl_6_	1	0.02	0.75	0.03	0.01	0.96
Cs_2_LiBiBr_6_	1	2.39	−1.54	4.35	1.43	0.95
Cs_2_CuBiBr_6_	1	−0.79	−1.71	0.96	0.65	0.95
Cs_2_AgSbCl_6_	1	−2.73	−2.88	1.39	1.56	0.94
Cs_2_AgSbBr_6_	1	0.76	1.33	1.90	2.67	0.93
Cs_2_NaBiCl_6_	1	3.92	4.41	6.33	1.63	0.92
Cs_2_InScBr_6_	1	−35.10	−27.99	11.93	11.89	0.92
Cs_2_NaBiBr_6_	1	−27.10	−21.48	9.95	8.50	0.91
Cs_2_AgBiCl_6_	1	0.03	0.07	4.29	1.60	0.90
Cs_2_NaBiI_6_	1	−117.54	−111.60	13.36	13.42	0.89
Cs_2_AgBiBr_6_	1	−2.43	−0.65	7.28	5.47	0.89
Cs_2_AgBiI_6_	1	−40.13	−30.79	11.44	10.95	0.88
Rb_2_NaBiCl_6_	1	−187.49	−183.23	14.52	14.47	0.88
Rb_2_NaBiBr_6_	1	−250.49	−241.73	15.55	15.36	0.87
Cs_2_KBiCl_6_	1	−147.24	−137.34	15.13	14.23	0.87
Rb_2_AgBiCl_6_	1	−171.96	−165.38	14.60	14.47	0.86
Rb_2_NaBiI_6_	1	−355.48	−352.12	16.03	16.13	0.86
Cs_2_KBiBr_6_	1	−188.13	−179.36	16.14	16.26	0.86
Rb_2_AgBiBr_6_	1	−204.13	−195.04	15.16	15.07	0.85
Cs_2_KBiI_6_	1	−265.28	−258.33	16.95	16.82	0.85
Cs_2_RbBiCl_6_	1	−274.39	−267.12	19.01	18.89	0.85
Rb_2_AgBiI_6_	1	−263.87	−256.04	15.42	15.38	0.84
Cs_2_TlAsBr_6_	2	1.21	2.75	7.82	7.55	0.91
Cs_2_InSbBr_6_	2	−7.37	−3.07	8.85	8.04	0.91
Cs_2_TlAsI_6_	2	−15.73	−12.92	9.44	9.25	0.89
Cs_2_TlSbBr_6_	2	−87.44	−81.01	14.27	14.13	0.88
Cs_2_InBiCl_6_	2	−76.19	−64.89	14.08	13.61	0.88
Rb_2_InSbCl_6_	2	−241.15	−233.09	17.81	17.83	0.88
Rb_2_TlAsBr_6_	2	−176.37	−172.43	16.72	16.58	0.87
Cs_2_TlSbI_6_	2	−98.53	−96.47	14.25	14.11	0.87
Cs_2_InBiBr_6_	2	−54.97	−50.79	12.83	12.85	0.87
Rb_2_InSbBr_6_	2	−185.63	−182.13	16.21	16.53	0.87
Cs_2_TlBiCl_6_	2	−148.13	−138.59	16.36	16.58	0.85
Cs_2_TlBiBr_6_	2	−145.46	−140.02	15.27	15.46	0.84
Rb_2_InBiCl_6_	2	−333.65	−334.60	18.97	18.37	0.84
Cs_2_TlBiI_6_	2	−158.16	−154.37	15.21	15.39	0.83

We classify HDPs based on the number of ns^2^ lone-pair cations on the B′ and B″ sites. Compounds where neither B′ nor B″ hosts an ns^2^ cation are assigned LP = 0. If exactly one of the B′ or B″ cations has an ns^2^ configuration, for example Bi^3+^, Sb^3+^, or As^3+^ on B″, the compound is assigned LP = 1. If both B′ and B″ host ns^2^ cations, for example In^+^ or Tl^+^ on B′ together with Bi^3+^/Sb^3+^/As^3+^ on B″, the compound is assigned LP = 2.

**Fig. 2:**
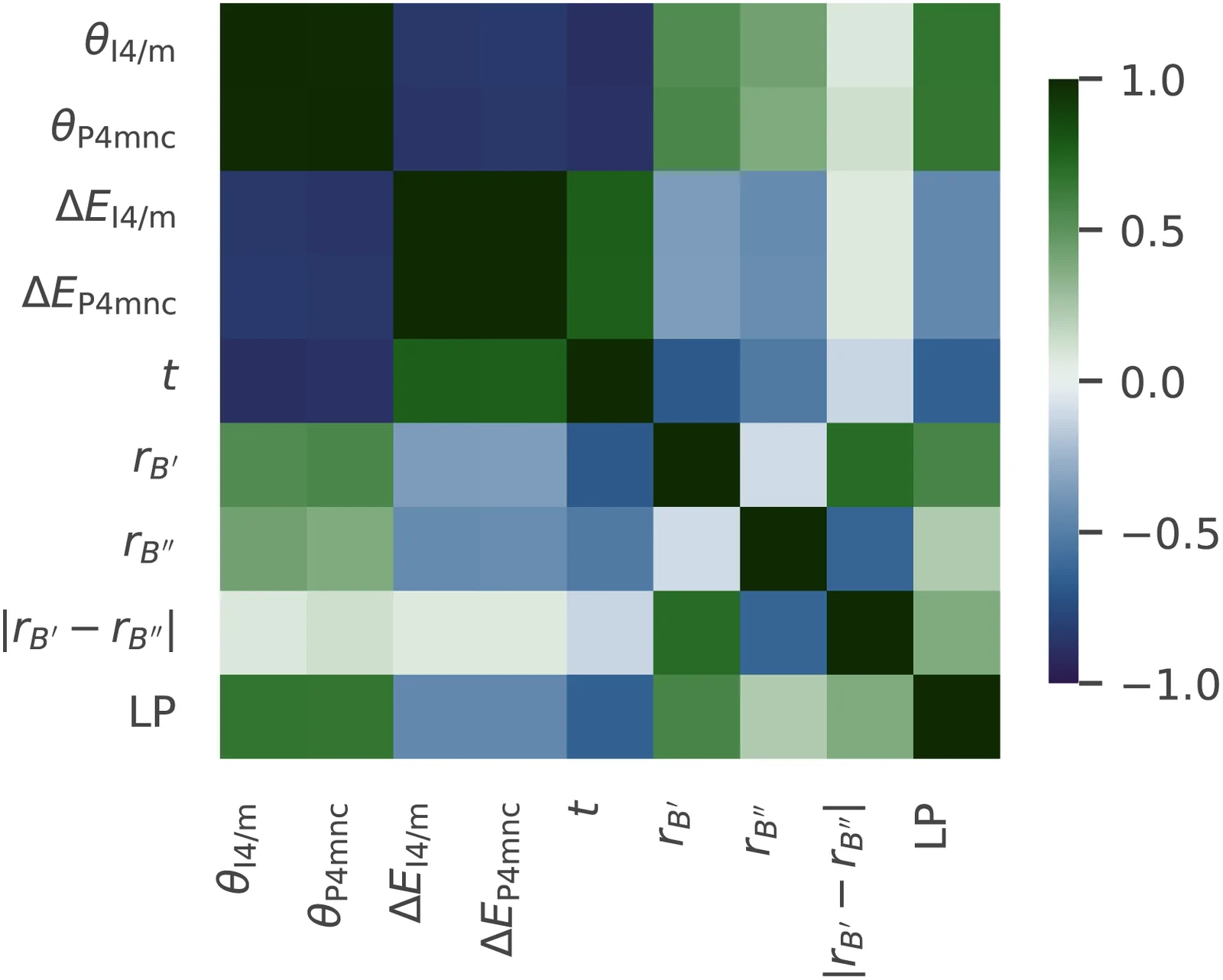
Pearson correlation matrix (*r*) for computed tilt angles and relative energies of the *I*4/*m* and *P*4/*mnc* phases, together with selected geometric descriptors (*t*, *r*_B′_, *r*_B″_, and |*r*_B′_ − *r*_B″_|) and the number of lone pairs (LP). Colors indicate *r* (−1 ≤ *r* ≤ 1).

To identify the key descriptors controlling octahedral tilting across the HDP dataset, we first examine correlations between the computed tilt angles and tilt energies and a set of chemical and geometric metrics. Fig. 2 shows the Pearson correlation matrix for the full dataset. A clear correlation is observed between the Goldschmidt tolerance factor *t* and both the magnitude of the tilt angles and the stabilization energy of the tilted phases: compounds with smaller *t* generally exhibit larger tilt angles and a stronger energetic preference for tilted structures. Related tolerance-factor trends have also emerged from previous studies on single perovskites.^52,53^ In contrast, the number of ns^2^ lone pairs shows a weaker direct correlation with tilting and energetics. However, LP is itself correlated with ionic-size descriptors and *t* across the present chemical space, indicating that the apparent LP-tilting trend largely reflects systematic compositional differences in ionic radii rather than an independent driving mechanism.

**Fig. 3:**
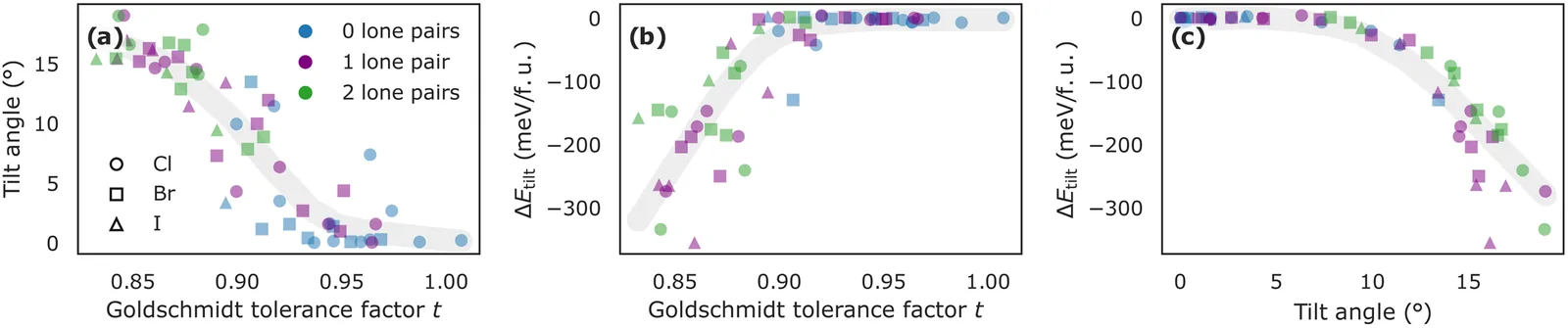
Relationships between structural distortion and energetic stability in the double-halide perovskite dataset: (a) tilt angle *vs.* Goldschmidt tolerance factor *t*, (b) Δ*E*_tilt_*vs. t*, and (c) Δ*E*_tilt_*vs.* tilt angle. Colors indicate the number of lone pairs (LP) and symbols denote the halide. The gray lines serve as a guide to the eye, indicating the overall trend.

These trends are further illustrated in Fig. 3. Here, we define the tilt energy Δ*E*_tilt_ = min(Δ*E*_*I*4/*m*_, Δ*E*_*P*4/*mnc*_) as the stabilization energy of the lowest-energy one-tilt tetragonal phase relative to the cubic phase. In Fig. 3a, the tilt angle increases systematically as *t* decreases, with only modest scatter between halides and lone-pair classes. Fig. 3b shows that Δ*E*_tilt_ becomes increasingly negative as *t* decreases, demonstrating that geometric mismatch provides a strong predictor for the stabilization of octahedral tilting. Finally, Fig. 3c shows a direct relationship between Δ*E*_tilt_ and tilt angle: larger tilts are generally associated with stronger energetic stabilization of the distorted phase.

**Fig. 4:**
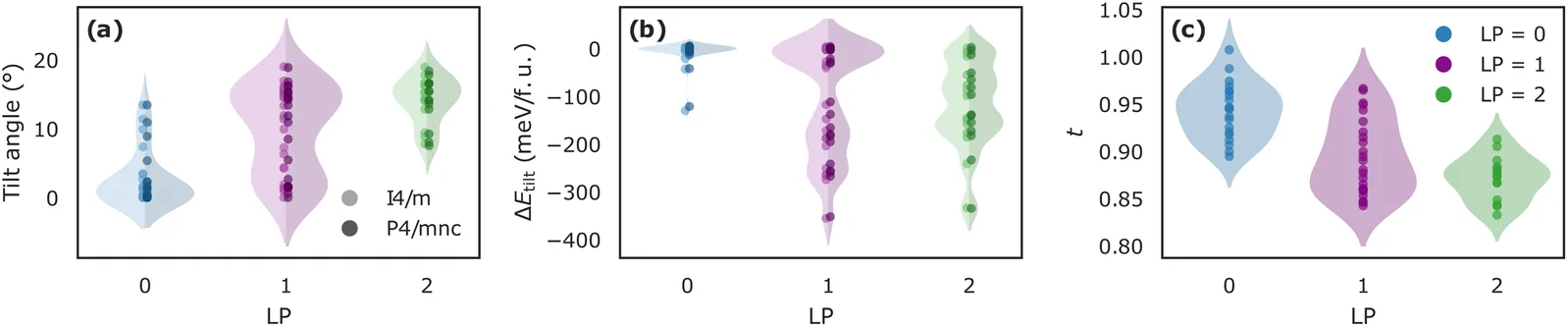
Distributions of structural, energetic, and geometric descriptors grouped by the number of ns^2^ lone pairs (LP). Violin plots show (a) octahedral tilt angles, (b) relative energies Δ*E*_tilt_, and (c) tolerance factors *t* for compounds with LP = 0, 1, and 2. Points indicate individual compounds.

To visualize these trends in a compact form, Fig. 4 shows the distributions of tilt angles, tilt energies, and *t* grouped by LP. While compounds with LP = 2 tend to display larger tilt angles and stronger stabilization of the tilted phases than compounds with LP = 0, Fig. 4c demonstrates that these groups also occupy different tolerance factor ranges. This indicates that the LP dependence is, to a significant extent, mediated by systematic differences in ionic radii that shift the tolerance factor away from the cubic ideal. Conversely, the LP = 0 group contains both nearly cubic materials, with *t* ≈ 1, and strongly tilted compounds where *t* significantly deviates from unity, consistent with a primarily geometric origin of the tilting instability.

Several compositions illustrate this point. For example, Cs_2_KInCl_6_, Rb_2_NaInCl_6_, and Rb_2_NaInBr_6_ contain alkali metals on the *B*′ site and exhibit pronounced tilting despite the absence of lone-pair cations. These compounds are characterized by tolerance factors substantially below unity, consistent with tilting driven by geometric mismatch and electrostatic packing effects. Within the LP = 1 group, a wide range of tilt angles and energies is observed, which again tracks variations in *t* rather than LP alone: materials such as Cs_2_AgBiI_6_ and Cs_2_KBiBr_6_ display large tilts and strongly negative Δ*E*_tilt_, while others such as Cs_2_AgBiBr_6_ exhibit comparatively weak stabilization of the tilted phase.

**Fig. 5:**
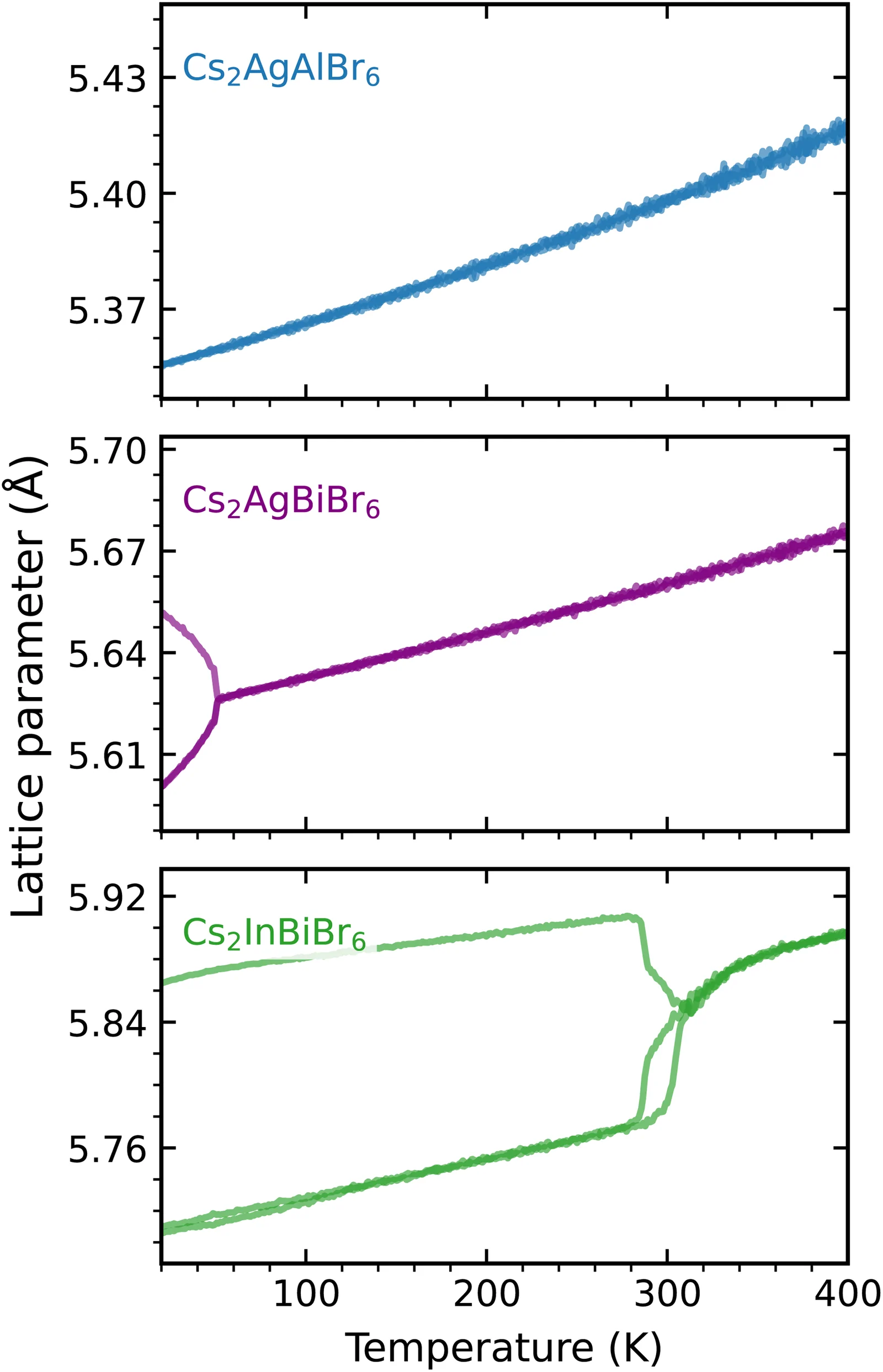
Temperature dependence of the lattice parameter for representative double-halide perovskites (Cs_2_AgAlBr_6_, Cs_2_AgBiBr_6_, and Cs_2_InBiBr_6_) obtained from cooling simulations. Discontinuities indicate structural phase transitions.

So far, we have only considered the relaxed structure of HDPs at 0 K. In lead- and tin-based halide perovskites, it has been shown that the degree of octahedral tilting at 0 K correlates with finite-temperature phase transition behavior.^35^ It is therefore useful to connect the 0 K tilting descriptors discussed above, namely tilt angles and tilt energies, to transition temperatures in representative HDPs. For this purpose, we select three representative compounds spanning the range of tilting tendencies in our dataset: Cs_2_AgAlBr_6_, Cs_2_AgBiBr_6_, and Cs_2_InBiBr_6_. These materials also span different lone-pair counts, LP = 0, 1, and 2, but, importantly, they differ systematically in their tolerance factors and 0 K tilting energetics. We perform MLIP-driven cooling simulations in the NPT ensemble using large supercells containing 40 000 atoms. The evolution of the pseudo-cubic lattice parameters with temperature is shown in Fig. 5.

For Cs_2_AgAlBr_6_, which is close to the cubic limit with negligible 0 K tilting, the lattice parameter varies smoothly over the full temperature range and no clear structural transition is observed. In contrast, Cs_2_AgBiBr_6_ and Cs_2_InBiBr_6_, which exhibit larger tilt angles and more negative tilt energies at 0 K, show clear abrupt changes in the temperature dependence of the lattice parameter, indicative of structural phase transitions. In Cs_2_AgBiBr_6_, a tetragonal-to-cubic transition is observed at about 50 K and in Cs_2_InBiBr_6_ it occurs at about 310 K. These observations support the general trend that stronger stabilization of tilted phases at 0 K, that is, more negative Δ*E*_tilt_ and larger *θ*, is associated with higher transition temperatures, consistent with a primarily geometric control of the tilting instability across the HDP family.

**Fig. 6:**
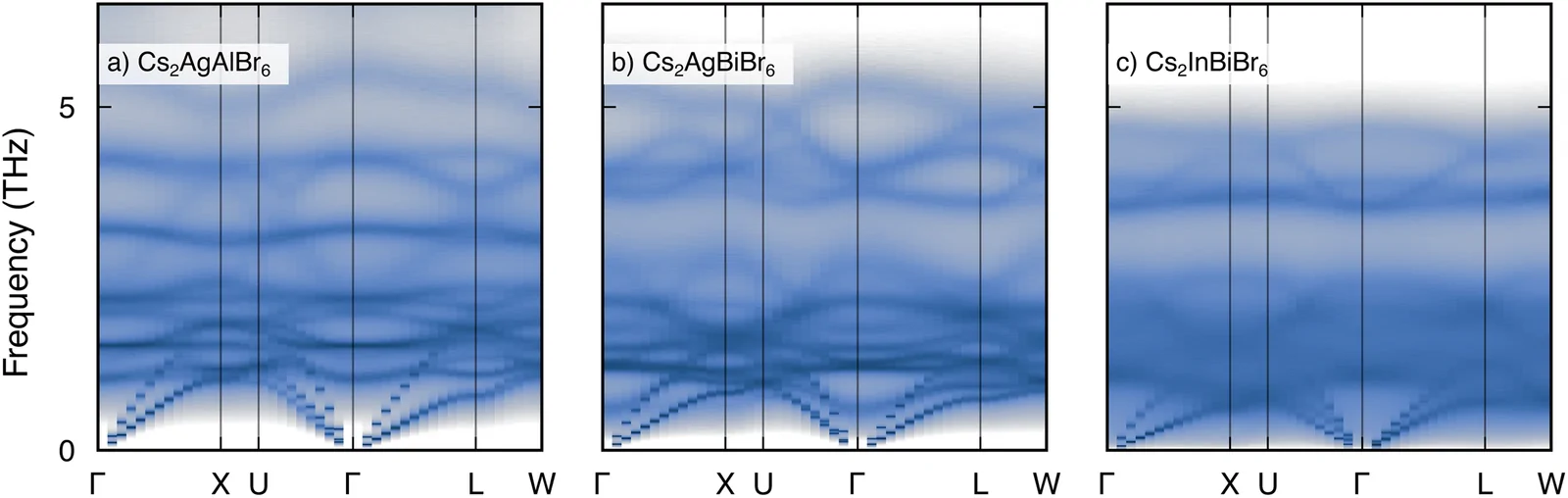
Phonon spectral energy density at 350 K for the cubic phase of (a) Cs_2_AgAlBr_6_, (b) Cs_2_AgBiBr_6_, and (c) Cs_2_InBiBr_6_ along high-symmetry directions in the Brillouin zone.

Beyond the average structural signatures in the lattice parameters, the finite-temperature vibrational spectra provide a complementary view of the lattice softness and anharmonicity. Fig. 6 shows the phonon SED at 350 K for Cs_2_AgAlBr_6_, Cs_2_AgBiBr_6_, and Cs_2_InBiBr_6_ along high-symmetry directions. The plotted path includes the Brillouin-zone boundary points associated with octahedral tilting instabilities in the cubic double-perovskite structure.^54^ For Cs_2_AgAlBr_6_, which remains close to the cubic limit with *t* ≈ 1 and negligible 0 K tilting, the SED exhibits relatively sharp dispersive features. In contrast, for Cs_2_AgBiBr_6_ and especially Cs_2_InBiBr_6_, which display larger tilt angles and stronger stabilization of tilted phases at 0 K, and correspondingly smaller *t*, the low-frequency features across the Brillouin zone become markedly broader and more diffuse. Such spectral broadening is consistent with increased anharmonicity and reduced phonon lifetimes, indicating a softer lattice and stronger anharmonicity.

Lattice softness and anharmonic lattice fluctuations have been widely discussed as contributing to the favorable optoelectronic properties and defect tolerance of lead-halide perovskites, for example through enhanced dielectric screening, polaron formation, and suppression of nonradiative recombination.^55,56^ In this context, our systematic evaluation of tilt angles and tilt energies provides a reference for comparing the relative strength of octahedral-tilting instabilities and associated trends in lattice softness across HDPs. While the quantitative impact on device performance will depend on detailed band structure and defect physics, the present dataset offers a framework for guiding materials selection. Similar geometric control of tilting *via* ionic-size mismatch is expected to apply in similar material families, for example oxide and chalcogenide perovskites.

## Conclusions

In this study, we have investigated octahedral tilting instabilities and the stability of one-tilt tetragonal phases in a chemically diverse set of halide double perovskites. By combining DFT calculations for the cubic and two symmetry-distinct tetragonal variants with statistical analysis of structural and chemical descriptors, we identify robust trends linking tilt angles and tilt stabilization energies to simple geometric metrics.

Across the explored chemical space, the Goldschmidt tolerance factor *t* emerges as the strongest predictor of both the magnitude of octahedral tilting and the energetic preference for tilted phases. Compounds with *t* close to unity generally remain nearly cubic with small tilt angles and weak stabilization of the tetragonal variants, whereas decreasing *t* is associated with larger tilt angles and increasingly negative tilt energies. While materials containing ns^2^ lone-pair cations tend to show stronger tilting on average, we find that this trend is largely mediated by systematic differences in ionic radii: lone-pair chemistry correlates with tilting primarily through its correlation with *t* within the present dataset. Importantly, pronounced tilting is also found in several LP = 0 compounds containing alkali metals on the B′ site, consistent with tilting driven by geometric packing and electrostatic considerations rather than lone-pair activity alone.

To connect the 0 K descriptors to finite-temperature behavior, we trained MLIPs and performed large-scale NPT cooling simulations for three representative compounds, Cs_2_AgAlBr_6_, Cs_2_AgBiBr_6_, and Cs_2_InBiBr_6_. The temperature evolution of the lattice parameters reveals clear phase-transition signatures for the more strongly tilted compounds, while Cs_2_AgAlBr_6_ remains close to the cubic limit over the investigated temperature range. Furthermore, SED analysis at 350 K shows progressively broader low-frequency features as *t* decreases, indicating increased anharmonicity and shorter phonon lifetimes. Together, these results demonstrate that tilt angles and tilt energies at 0 K provide a useful qualitative indicator of finite-temperature lattice softness and transition behavior, with geometric packing, captured by the tolerance factor, serving as the dominant control parameter across the halide double perovskite family.

## Conflicts of interest

There are no conflicts to declare.

## Data Availability

DFT input files, structures, NEP models, as well as the DFT databases used to train these models, are available on Zenodo at https://doi.org/10.5281/zenodo.18841767.
